# Vitamin K Vitamers Differently Affect Energy Metabolism in IPEC-J2 Cells

**DOI:** 10.3389/fmolb.2021.682191

**Published:** 2021-05-24

**Authors:** Chiara Bernardini, Cristina Algieri, Debora La Mantia, Fabiana Trombetti, Alessandra Pagliarani, Monica Forni, Salvatore Nesci

**Affiliations:** ^1^Department of Veterinary Medical Science, University of Bologna, Ozzano Emilia, Italy; ^2^Health Sciences and Technologies—Interdepartmental Center for Industrial Research (CIRI-SDV), Alma Mater Studiorum—University of Bologna, Bologna, Italy

**Keywords:** vitamin K, mitochondria, oxidative phosphorylation, glycolysis, IPEC-J2, ATP

## Abstract

The fat-soluble vitamin K (VK) has long been known as a requirement for blood coagulation, but like other vitamins, has been recently recognized to play further physiological roles, particularly in cell development and homeostasis. Vertebrates cannot *de novo* synthesize VK, which is essential, and it can only be obtained from the diet or by the activity of the gut microbiota. The IPEC-J2 cell line, obtained from porcine small intestine, which shows strong similarities to the human one, represents an excellent functional model to *in vitro* study the effect of compounds at the intestinal level. The acute VK treatments on the bioenergetic features of IPEC-J2 cells were evaluated by Seahorse XP Agilent technology. VK exists in different structurally related forms (vitamers), all featured by a naphtoquinone moiety, but with distinct effects on IPEC-J2 energy metabolism. The VK1, which has a long hydrocarbon chain, at both concentrations (5 and 10 μM), increases the cellular ATP production due to oxidative phosphorylation (OXPHOS) by 5% and by 30% through glycolysis. The VK2 at 5 μM only stimulates ATP production by OXPHOS. Conversely, 10 μM VK3, which lacks the long side chain, inhibits OXPHOS by 30% and glycolysis by 45%. However, even if IPEC-J2 cells mainly prefer OXPHOS to glycolysis to produce ATP, the OXPHOS/glycolysis ratio significantly decreases in VK1-treated cells, is unaffected by VK2, and only significantly increased by 10 μM VK3. VK1, at the two concentrations tested, does not affect the mitochondrial bioenergetic parameters, while 5 μM VK2 increases and 5 μM VK3 reduces the mitochondrial respiration (i.e., maximal respiration and spare respiratory capacity). Moreover, 10 μM VK3 impairs OXPHOS, as shown by the increase in the proton leak, namely the proton backward entry to the matrix space, thus pointing out mitochondrial toxicity. Furthermore, in the presence of both VK1 and VK2 concentrations, the glycolytic parameters, namely the glycolytic capacity and the glycolytic reserve, are unaltered. In contrast, the inhibition of glycoATP production by VK3 is linked to the 80% inhibition of glycolysis, resulting in a reduced glycolytic capacity and reserve. These data, which demonstrate the VK ability to differently modulate IPEC-J2 cell energy metabolism according to the different structural features of the vitamers, can mirror VK modulatory effects on the cell membrane features and, as a cascade, on the epithelial cell properties and gut functions: balance of salt and water, macromolecule cleavage, detoxification of harmful compounds, and nitrogen recycling.

## Introduction

Vitamin K (VK) was originally recognized as a component in blood clotting, being a cofactor for vitamin K–dependent carboxylase, which facilitates the post-translational modification of glutamic acid to γ-carboxy-glutamic acid residues in selected proteins (Suttie, 1985). The relevant recognized VK role in coagulation, hence the symbol K, from the German term “Koagulation,” promptly enrolled VK among micronutrients.

Even if partially provided by gut microbiota, VK requires dietary uptake, even if the required amounts are still undefined. The intestinal absorption of VKs follows the dietary lipid pattern (Shearer et al., 2012).

**FIGURE 1:**
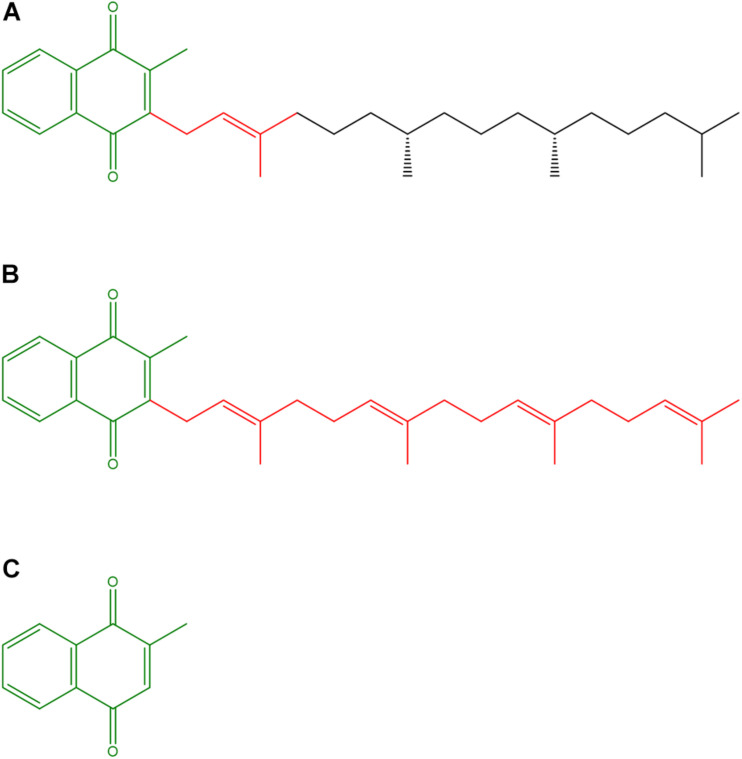
Structures of vitamin K vitamers. **(A)** Phylloquinone or Vitamin K1; **(B)** Menaquinone-4 or Vitamin K2; **(C)** Menadione or Vitamin K3. The 2-methyl-1,4-naphthoquinone nucleus and the (poly)isoprenoid side chains are highlighted in olive and red, respectively.

The VK family consists of structurally similar naphthoquinones (Figure 1), which, due to their lipophilicity, can easily cross cell membranes. VK naturally exists in two forms, namely, phylloquinone (VK1), which bears a phytyl side chain, mainly comes from vegetables and constitutes more than 90% of dietary VKs, and menaquinones (VK2), which exist in multiple structures, mainly come from bacterial synthesis in the gut, and occur in fermented foods (Schwalfenberg, 2017). The extent to which endogenous VK2 production contributes to the daily VK requirement is still unknown (Shearer et al., 2012). Accordingly, the VK2 form menaquinone-4 can be synthesized from VK1, which is absorbed in the small intestine, and represents the most abundant VK form in tissues (Okano et al., 2008). The VK homologs are characterized by a 2-methyl-1,4-naphthoquinone nucleus and a polyisoprenoid side chain at the 3-position (Figure 1). In VK2 vitamers the side chain varies in both length and saturation degree (Tsugawa and Shiraki, 2020). The VK2 basic structure, which has a side chain with four double bonds, is similar to Coenzyme Q_10_ (CoQ_10_), involved as an electron carrier in mitochondrial respiration, but has a shorter hydrophobic side chain whose four prenyl units confer higher hydrophilicity. Finally, menadione, or VK3, lacks the hydrocarbon side chain and is considered as a metabolite or a provitamin (Schwalfenberg, 2017).

Over the last two decades, roles and action mechanisms of VKs other than the involvement in γ-carboxylation, a post-translational modification which modulates the function of various proteins, have been discovered. Recently, VKs were found to play a relevant role in extrahepatic metabolism, such as in bone and blood vessels, in energy metabolism (Tsugawa and Shiraki, 2020), and in counteracting inflammation, as VK deficiency has been associated with human diseases with inflammatory background. Until very recently, VKs were considered capable of playing a role against colorectal cancer and other cancer types (Schwalfenberg, 2017) by inhibiting the cell proliferation, including an active induction of the cell cycle arrest, and to induce apoptosis in different human gastrointestinal cancer cells (Orlando et al., 2015). VKs also improve cognitive function in elderly patients and decrease the risk of diabetes (Schwalfenberg, 2017). At least some of these emerging roles may also be related to the VK involvement in signaling as modulators of selected nuclear receptors. Accordingly, VK2 (menaquinone-4) binds to the transcription factor SXR/PXR, thus acting like a hormone like other lipophilic vitamins. Therefore, its effects are widely spread in body systems, and it is recognized to increase longevity (Gordeladze, 2017). VK2 can also bind the 17β-Hydroxysteroid dehydrogenase type 4 (17β-HSD4) and activate protein kinase A (PKA), but the mechanisms of binding and enzyme regulation remain unknown (Azuma and Inoue, 2019).

The CoQ_10_-like structure of VK2 has raised hopes to exploit VK to treat some mitochondrial defects (Vos et al., 2012). However, the possible replacement of CoQ_10_ by VK2 in the respiratory chain, as well as the putative role of vitamins K3 and C to improve ATP production by reducing cytochrome *c* through two coupled redox cycles (Ivanova et al., 2018), are still a matter of debate. Unexpectedly, VK2 was a poorly efficient respiratory substrate in human cells *in vitro* (Cerqua et al., 2019). VK3 is potentially toxic and able to counteract some cancer types (Schwalfenberg, 2017). Interestingly, VK3 affects the redox status of thiols, can induce oxidative stress in cancer cells, and seems the most efficient VK form in combination with vitamin C to restore oxidative phosphorylation (Ivanova et al., 2018).

Up to now, VK involvement in the bioenergetics of enterocytes, which are not only directly involved in VK absorption but also are in proximity with gut microbiota which provide VK2, has not been explored. The IPEC-J2 cell line (Vergauwen, 2015), initially established in 1989 and obtained from the small intestine of the pig, which shows anatomical and physiological similarities to humans, has been selected as *in vitro* model to investigate the action mechanisms at the biochemical and molecular level of a variety of compounds on mammalian intestine (Wu et al., 2019). Due to their features, IPEC-J2 cells provide an excellent *in vitro* model to investigate the effects of VKs on cell bioenergetics. This cell line is neither transformed nor tumorigenic and reproduces the human physiology features more closely than any other cell line of non-human origin. Of note, this cell line guarantees the reproducibility of the results since it maintains the differentiated characteristics and exhibits strong similarities to primary cell cultures. As far as we are aware, only a few studies approached cell bioenergetics in this cell line under normal conditions (Tan et al., 2015; Bernardini et al., 2021), highlighting that these cells mirror the known behavior of intestinal cells, since they preferentially derive energy from glucose plus glutamine than from glucose alone. Enterocytes mainly use glycolysis to provide metabolic precursors to the liver, while mitochondrial respiration provides the main energy source (Nesci, 2017). IPEC-J2, as well as IPEC-J1, have the typical differentiation of an enterocyte, which is independent of the culture system. The aerobic environment can start the initial proliferation and sequential differentiation of intestinal epithelial cells and progeny loss (Nossol et al., 2011). Enterocyte mitochondrial function is important for gut permeability. Accordingly, mitochondrial uncoupling increases intestinal permeability, generating local and systemic inflammation, which is associated with the development of inflammatory bowel diseases (Bórquez et al., 2020).

This study, which focuses on VK effects on IPEC-J2 cells, aims at deepening the knowledge on the multiple VK roles and on the responses of the energy machinery to these quinone compounds. IPEC-J2 cells, which exhibit strong similarities to primary intestinal epithelial cells, may address studies on the most suitable VK form to modulate enterocyte bioenergetics. Accordingly, structural differences among VK vitamers may be relevant to produce different effects. Moreover, this study, by providing clues on the putative modulatory role of VKs on cell bioenergetics, may open further therapeutic perspectives of these fascinating micronutrients.

## Materials and Methods

### Chemicals

α-Phylloquinone (Vitamin K1), Menaquinone-4 (Vitamin K2), and Menadione (Vitamin K3) were purchased from Cayman Chemical. Seahorse XF Assay Kits and Reagents were purchased from Agilent. All other chemicals were reagent grade and used without purification. Quartz double-distilled water was used for all reagent solutions, except when otherwise stated.

### Cell Culture

The non-transformed cell line IPEC-J2 was purchased from the “Deutsche Sammlung von Mikroorganismen und Zellkulturen GmbH” (DSMZ). Cells were cultured in Dulbecco’s Modified Eagle Medium (DMEM) (4.5 g/l glucose) added with 10% of fetal bovine serum (FBS, Life Technologies) and 1× antibiotic-antimycotic solution (Life Technologies) in a 5% CO_2_ atmosphere at 37°C. Cultures were split weekly in T25 [0.5 × 10^6^ or T75 (1.5 × 10^6^) culture flasks (Corning-Becton Dickinson and Company Becton Drive, Franklin Lakes, NJ, United States). For cryopreservation, 1.5 × 10^6^ were resuspended in 1 ml of freezing medium (DMEM added with 20% FBS, 1× antibiotic-antimycotic solution, and 10% DMSO). The cryovials were placed in a freezing box and stored at -80°C overnight. For long-term storage, the cryovials were transferred into a cryogenic biobank.

### Cellular Bioenergetics

The Seahorse XFp analyzer (Agilent) was used to simultaneously measure oxygen consumption rate (OCR), an index of cell respiration (pmol/min), and extracellular acidification rate (ECAR), an index of glycolysis (mpH/min). IPEC-J2 cells (1 × 10^4^ / well) were grown in XFp cell culture miniplates (Agilent) for 24 h. On the experiment day, IPEC-J2 cells were switched to freshly made Seahorse XF DMEM medium pH 7.4 supplemented with 10 mM glucose, 1 mM sodium pyruvate, and 2 mM L-glutamine, in the absence (Control) and in the presence of 5 or 10 μM of each VK vitamer (VK1, VK2 or VK3) under study. The plates were incubated at 37°C in air for 45 m in before measuring OCR and ECAR by the adequate programs (ATP Rate Assay, Cell Mito Stress Test and Cell Energy Phenotype Test). The injection ports of XFp sensor cartridges, which were hydrated overnight with XF calibrant at 37°C, were loaded with 10× concentration of inhibitors according to the instructions provided by Seahorse XFp ATP Rate Assay, Cell Mito Stress Test, and Cell Energy Phenotype Test. The final concentration used for ATP Rate Assay were 1.5 μM oligomycin (port A) and 0.5 μM rotenone (Rot) plus 0.5 μM antimycin A (AA) (port B). For Cell Mito Stress Test the final concentrations were 1.5 μM oligomycin (olig) (port A), 1.0 μM Carbonyl-cyanide-4-(trifluoromethoxy) phenylhydrazone (FCCP) (port B), and 0.5 μM rotenone plus antimycin A (port C), while for the Cell Energy Phenotype Test the final concentrations were 1.5 μM oligomycin plus 1.0 μM FCCP (port A). All the analysis were run at 37°C. All data were analyzed by WAVE software; OCR and ECAR values were normalized to the total number of cells per well. All parameter values were calculated per well, according to the manufacturer’s instructions. Both ATP Rate Assay, Mito Stress Test, and Cell Energy Phenotype Test were carried out three times in independent experiments (Marcoccia et al., 2021).

The ATP Rate Assay provides the bioenergetic parameters currently used to characterize the cellular ATP production, namely ATP production rate, related to the conversion of glucose to lactate in the glycolytic pathway (glycoATP Production Rate) and to the mitochondrial OXPHOS (mitoATP Production Rate). Accordingly, the ratio between mitoATP Production Rate and glycoATP Production Rate (ATP Rate Index) is currently considered as a valuable parameter to detect changes and/or differences in the metabolic phenotype (a ratio >1 means mainly OXPHOS pathway; a ratio <1 means mainly glycolytic pathway).

The Mito Stress Test enables the characterization of cell respiration by the following parameters: basal respiration, detected as baseline OCR before oligomycin addition; minimal respiration measured as OCR in the presence of oligomycin; and maximal respiration evaluated as OCR after FCCP addition. The so-called proton leak, which corresponds to the difference between the basal respiration and the respiration in the presence of oligomycin (minimal respiration), indicates the re-entry of H^+^ in the intermembrane space independently of the F_1_F_*O*_-ATP synthase. The non-mitochondrial respiration, evaluated as OCR in the presence of rotenone plus antimycin A (respiratory chain inhibitors), was subtracted from all the above parameters. The ATP turnover or oligomycin-sensitive respiration was obtained from the difference between the basal respiration and the minimal respiration (OCR in presence of oligomycin). Finally, the difference between the maximal and the basal respiration provided the spare capacity, which represents the ability to respond to an increased energy demand and can be considered as a measure of the flexibility of the OXPHOS machinery (Bernardini et al., 2021).

The simultaneous measurement of mitochondrial respiration and glycolysis was carried out by the Cell Energy Phenotype Test under baseline and stressed conditions, the latter after simultaneous addition of oligomycin and FCCP. Oligomycin inhibits the mitochondrial ATP production by the F_1_F_*O*_-ATP synthase and the cell compensates the failed OXPHOS by increasing the glycolysis rate, while the dissipation of the electrochemical gradient of H^+^ in mitochondria by the ionophore FCCP drives the highest oxygen consumption (uncoupled respiration). The assay allows to evaluate two main parameters of cell energy metabolism, known as metabolic phenotypes (baseline and stressed phenotype) and metabolic potential. The baseline phenotype is featured by the OCR and ECAR values in cells under the starting condition in the presence of substrates. The stressed phenotype is shown by the OCR and ECAR values in cells after addition of stressor compounds (oligomycin plus FCCP). The metabolic potential is the ability to increase energy production *via* respiration and glycolysis and it is defined as the % increase of stressed phenotype over baseline phenotype of OCR and ECAR (Bernardini et al., 2021).

### Statistical Analysis

Statistical analyses were performed by SIGMASTAT software. Each treatment was replicated three or eight times (viability test) in three independent experiments. Data were analyzed by the Student’s *t*-test, or by one-way analysis of variance (ANOVA) followed by Students–Newman–Keuls test when *F* values indicated significance (*P* ≤ 0.05) was applied. Percentage data were *arcsin*-transformed before statistical analyses to ensure normality.

## Results

### Intracellular ATP Production

**FIGURE 2:**
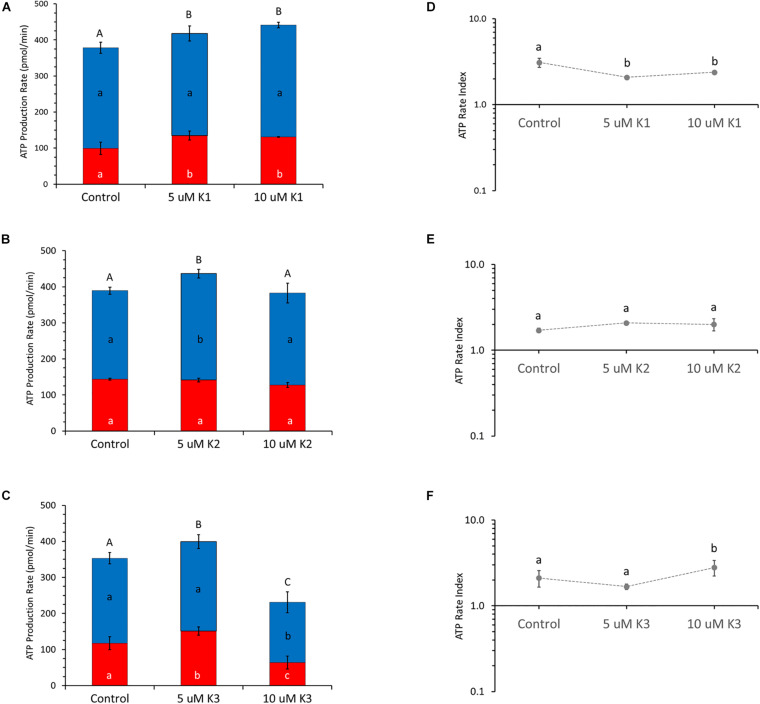
Effect of VKs on the real-time ATP production rate in IPEC-J2 cells. Evaluation of ATP production rate by mitochondrial OXPHOS (blue) (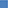) or by glycolysis (red) (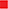) in VK1 **(A)**, VK2 **(B)**, and VK3 **(C)**-treated cells. The ATP rate index, calculated as the ratio between the mitochondrial ATP production rate and the glycolytic ATP production rate, is shown on the y-axis (logarithmic scale) in IPEC-J2 treated with 0 (Control), 5, and 10 μM VK1 **(D)**, VK2 **(E)**, and VK3 **(F)**. Data, expressed as column chart [**(A,B,C)** plots] and points [**(D,E,F)** plots], represent the mean ± SD (vertical bars) from three experiments carried out on distinct cell preparations. Different lower-case letters indicate significantly different values (*P* ≤ 0.05) among treatments (0, 5, 10 μM) in the same metabolic pathway; different upper-case letters indicate different values (*P* ≤ 0.05) among treatments in ATP production rates due to sum of OXPHOS plus glycolysis.

The cellular ATP level produced by OXPHOS and glycolysis in the presence of the different VK vitamers are shown in Figure 2 by OCR and ECAR values, respectively, under basal metabolic conditions. The calculation of the mitoATP and glycoATP production rate (Figure 2), obtained by injecting oligomycin to inhibit mitochondrial ATP synthesis and then rotenone plus antimycin A to block mitochondrial respiration, highlight that IPEC-J2 cells are characterized by an oxidative metabolism. VK1 induces an increase in total ATP production at both the concentrations tested (5 and 10 μM). This increase is due to a significant glycoATP production activation, whereas the mitoATP production is unaffected (Figure 2A). Conversely, VK2 does not affect the glycoATP production. The total ATP production is only 20% increased by 5 μM VK2 with respect to the control, due to an enhanced mitochondrial ATP synthesis (Figure 2B). VK3 shows a concentration-dependent effect on intracellular ATP production by increasing the glycolysis by 30%without modifying the mitochondrial activity at 5 μM, whereas at 10 μM it reduces by 50% the glycoATP and the mitoATP synthesis by 30% (Figure 2C). Even if all the VK vitamers can modify ATP production, the results highlight that the ratio between mitoATP Production Rate and glycoATP Production Rate (ATP Rate Index) is always > 1. In detail, 5 and 10 μM VK1 decrease the propensity to produce mitoATP with respect to the control (Figure 2D), whereas this effect is not shown by VK2 which does not modify the ATP rate index (Figure 2E). In IPEC-J2 cells treated with 10 μMVK3, the residual ATP production mainly relies on mitochondrial oxidative metabolism (Figure 2F).

### Cellular Respiration

**FIGURE 3:**
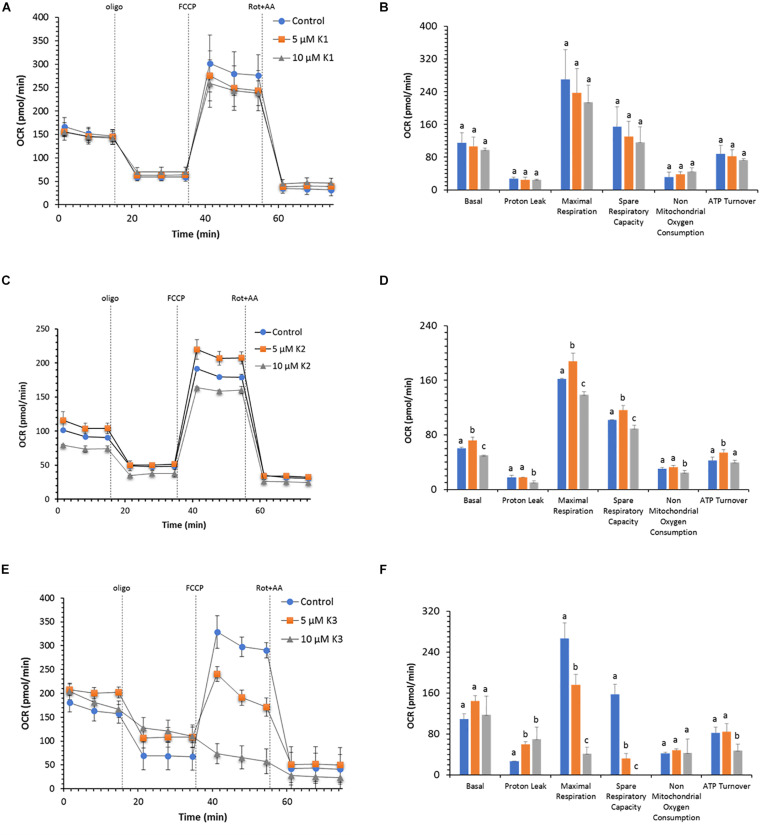
Effect of VKs on the mitochondrial respiration profile in IPEC-J2 cells. Oxygen consumption rate (OCR) at two concentrations (5 and 10 μM) of VK1 **(A)**, VK2 **(C)**, and VK3 **(E)** under basal respiration conditions and after the addition of 1.5 μM oligomycin (olig), 1.0 μM FCCP and a mixture of 0.5 μM rotenone plus antimycin A (rot+AA). Inhibitor injections are shown as dotted lines. Mitochondrial parameters (basal respiration, ATP production, proton leak, maximal respiration, spare respiratory capacity, non-mitochondrial oxygen consumption, ATP turnover) in VK absence (blue) (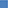) or in the presence of 5 μM (orange) (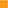), or 10 μM (gray) (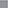) VK1 **(B)**, VK2 **(D)**, and VK3 **(F)**. Data expressed as points **(A,C,E)** and column chart **(B,D,F)** represent the mean ± SD (vertical bars) from three experiments carried out on different cell preparations. Different letters indicate significant differences (*P* ≤ 0.05) among treatments within the same parameter.

The profile and function of cell respiration of IPEC-J2 cells treated with VKs are shown in Figure 3. The key parameters of cell metabolism were obtained from functional metabolic data as detailed in the Materials and mMethods section. The results show that OCR values in the presence of both VK1 concentrations tested are not different from control ones (Figures 3A,B). VK1 does not modify the basal respiration and the proton leak; consequently, the calculated ATP turnover is not affected. Therefore, the coupling efficiency in the presence and in the absence of VK1 is about 0.80 a.u. (the maximal value of 1.0 a.u. is obtained when all the basal respiration is sensitive to oligomycin). The IPEC-J2 OCR in the presence of FCCP (maximal respiration) shows a twice higher value than basal OCR. The spare respiratory capacity, which defines the cell propensity to adjust cell bioenergetics to fulfill the increased energy demand, constantly attains a 50% OCR value of the FCCP-stimulated OCR, irrespective of the VK1 presence (Figure 3B).

VK2 has a concentration-dependent effect on the OCR (Figure 3C). Accordingly, 5 μM VK2 increases the basal, maximal, and spare capacity OCR, as well as ATP turnover with respect to the control. Conversely, at 10 μM VK2 the effect on the same parameters is inhibitory (Figure 3D). Even if the basal and ATP turnover is higher in presence of 5 μM VK2 and lower at 10 μM VK2, with respect to the control, the coupling efficiencies values in the presence of VK2 (0.77 a.u.) are not different from those of the control. Likewise, on comparing the maximal respiration and the spare respiratory capacity in the absence or in the presence of the two VK2 concentrations, significantly different OCR values are shown. However, the OCR values of the maximal respiration and spare respiratory capacity in all treatments (0.0, 5.0, and 10 μM VK2) are of the same extent. In detail, the maximal respiration is always twice higher than the basal respiration, whereas for the latter the OCR activity is increased by 50% with FCCP (Figure 3D).

As shown in Figure 3E, the respiratory profile of IPEC-J2 cells decreases with increasing VK3 concentrations. Even if the basal respiration is apparently unaffected by VK3 (Figure 3F), the coupling efficiency is halved by 10 μM VK3 as a result of an increase in proton leak. In addition, IPEC-J2 cells treated with 5 μM VK3 do not show any respiration stimulation by FCCP, while when these cells are treated with 10 μM VK3, not only do they not show any increase in OCR in response to FCCP, but also the OCR after FCCP addition is lower than the basal OCR. Consistently, a strong inhibition of respiration with strongly decreased or even abolished spare respiratory capacity is detected in the presence of 5 and 10 μM VK3, respectively.

### Extracellular Acidification

**FIGURE 4:**
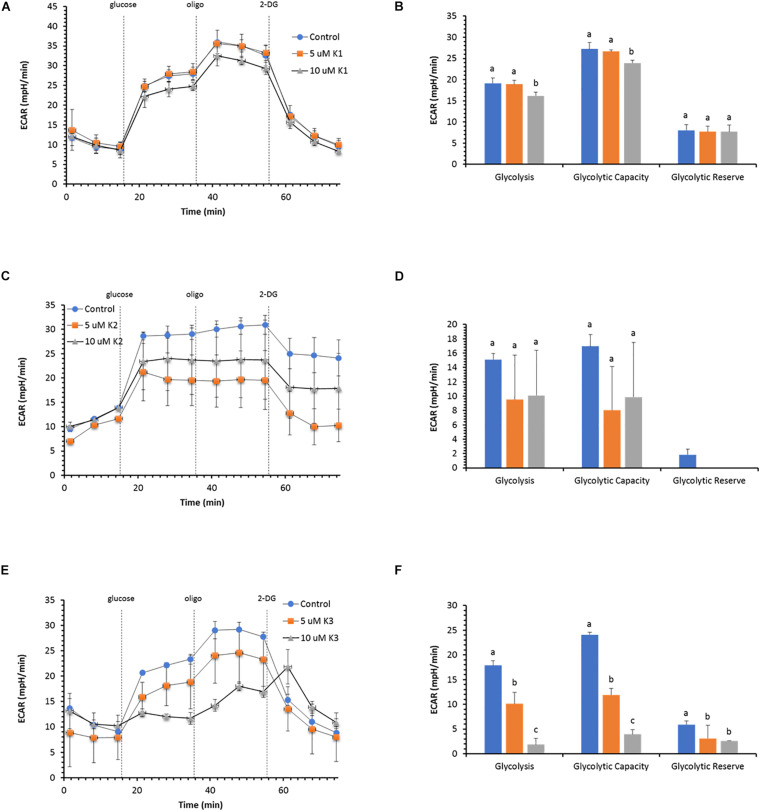
Effect of VKs on the glycolytic function in IPEC-J2 cells. Extracellular acidification rate (ECAR) at two concentrations (5 and 10 μM) of VK1 **(A)**, VK2 **(C)**, and VK3 **(E)** under basal conditions and after the addition of 10 mM glucose, 5 μM oligomycin (olig), and 50 mM 2-DG. Injections are shown as dotted lines. The three parameters of the glycolytic function: (glycolysis, glycolytic capacity, and glycolytic reserve) in VK absence (blue) (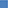), or in the presence of 5 μM (orange) (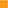), or 10 μM (gray) (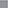) VK1 **(B)**, VK2 **(D)**, and VK3 **(F)**. Data expressed as points **(A,C,E)** and column chart **(B,D,F)** represent the mean ± SD (vertical bars) from three experiments carried out on different cell preparations. Different letters indicate significant differences (*P* ≤ 0.05) among treatments within the same parameter.

The glycolytic function in IPEC-J2 cells evaluated as extracellular acidification rate (ECAR) does not show any change in the presence of the two VK1 concentrations tested (Figure 4A). The key parameters of glycolytic flux, i.e., Glycolysis and Glycolytic Capacity, attain the same value in the presence or in the absence of 5 μM VK. Conversely, 10 μM VK1 inhibits both parameters by 15% (Figure 4B). However, the Glycolytic Reserve, defined as the difference between Glycolytic Capacity and Glycolysis, is unaffected by 10 μM VK1, since the two parameters show a proportional decrease in ECAR with respect to the control (Figure 3B).

Also, VK2 does not substantially modify the glycolytic profile of IPEC-J2 cells (Figure 4C), but in this case the glycolytic reserve disappears, since the Glycolysis rate has the same ECAR value as the Glycolytic Capacity (Figure 4D).

VK3 shows an inhibitory action on ECAR with a concentration-dependent effect (Figure 4E). The Glycolysis and the Glycolytic Capacity are inhibited by 50 and 13% with 5 and 10 μM VK3, respectively. Conversely, the decrease in the Glycolytic Reserve is independent of the VK3 concentration tested (Figure 4F).

### Bioenergetic Phenogram

**FIGURE 5:**
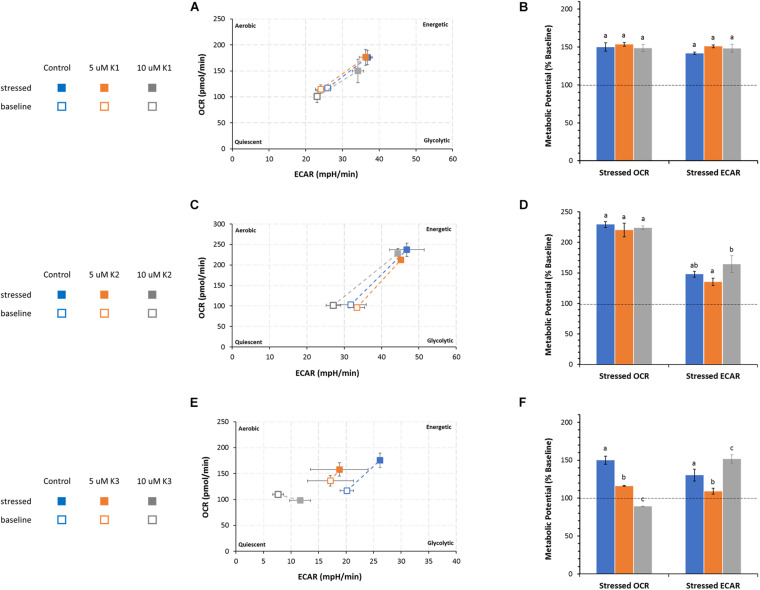
Effect of VKs on the energy map of IPEC-J2 cells. Baseline (empty squares) and stressed (full squares) phenotypes of IPEC-J2 cells in VK absence (control, blue squares) and in the presence of 5 μM, orange squares) or 10 μM (gray squares) VK1 **(A)**, VK2 **(C)**, and VK3 **(E)**. The Metabolic potential in “Stressed OCR” and “Stressed ECAR” is expressed as % “Baseline OCR” and “Baseline ECAR” (dashed horizontal line), in VK absence (blue) (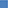) or in the presence of 5 μM (orange) (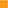), or 10 μM (gray) (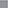) VK1 **(B)**, VK2 **(D)**, and VK3 **(F)**. Data expressed as points **(A,C,E)** and column chart **(B,D,F)** represent the mean ± SD (vertical and where present also horizontal bars) from three experiments carried out on distinct cell preparations. Different letters indicate significant differences (*P* ≤ 0.05) among treatments within the same parameter.

The cell energy production of IPEC-J2 cells detected by OCR and ECAR in the presence or in the absence of VKs is shown as metabolic phenotype under normal (baseline) and stressed conditions (Figure 5). The treatment with oligomycin and FCCP mixture, known as mitochondrial stressors, provides the phenogram which illustrates the relative baseline and stressed phenotype, and the response of the metabolic potential (expressed as % baseline) of IPEC-J2 cells after treatment with VKs.

According to the method adopted, the metabolic potential indicates the cell’s ability to meet an energy demand by mitochondrial respiration and glycolysis.

Irrespective of the presence of VK1, IPEC-J2 cells show an increase in the utilization of both pathways (mitochondrial respiration and glycolysis) (Figure 5A) under stressed conditions without any significant difference in the metabolic potential in the presence or absence of VK1 (Figure 5B).

The IPEC-J2 phenogram under starting assay conditions (baseline phenotype) in the presence of substrates and without stressors (oligomycin plus FCCP) do not show any difference in the metabolic pathways between control and VK2 treated cells. The same results are detected under stressed conditions irrespective of the presence of VK2, even if in this case the cells undergo a metabolic switch toward an energetic phenotype (Figure 5C). The cell energy demand is satisfied by an increase in OCR. The metabolic potential primarily relies on an efficient mitochondrial energy metabolism. No difference in OCR values between the VK2-treated IPEC-J2 cells and the control under stressed conditions is detectable (Figure 4D). The two VK2 concentrations tested do not modify the glycolytic activity stimulated by stressor compounds with respect to the control, even if the stressed ECAR, expressed as metabolic potential, in 5 μM VK2-treated IPEC-J2 cells is lower than that in 10 μM VK2-treated cells (Figure 5D).

VK3 affects the IPEC-J2 cells’ energy production, highlighting an energy map which shows an inhibited aerobic metabolism (Figure 5E). Stressed OCR and ECAR in the 5 μM VK3-treated IPEC-J2cells are inhibited by 23 and 16%, respectively. The treatment with 10 μM K3 makes IPEC-J2 cells shift their energy production mode from OXPHOS to glycolysis (Figure 5F). In these cells the stressed OCR is 11% lower than baseline OCR, whereas the metabolic potential of glycolysis (ECAR) increases by 50% when compared to baseline metabolism (Figure 5F). However, the baseline OCR/ECAR ratio in VK3-treated IPEC-J2 cells is > 4. This means that, other than acidification from glycolysis, the ECAR values in VK3-treated IPEC-J2 cells include the contribution of the mitochondrial acidification by CO_2_ production by Krebs cycle. Accordingly, in aqueous media CO_2_ forms the weak acid H_2_CO_3_.

## Discussion

As far as we are aware, up to now the effect of the three VK vitamers on the bioenergetic metabolism of enterocytes has been poorly investigated. The IPEC-J2 cell line provides an excellent model to evaluate in detail the mechanism(s) involved in cell energy production. These cells also represent a good translational model to study fat-soluble vitamin effects on cellular metabolism in the gut. IPEC-J2 cells rely on oxidative metabolisms to supply the energy required by the physiological functions (Vaugelade et al., 1994; Bernardini et al., 2021).

The enhancement of ATP production by IPEC-J2 cells is the most relevant bioenergetic effect of VK1, which primarily contributes to stimulate the glycolytic pathway at both the concentrations tested. Indeed, the modification of ATP rate index highlights that VK1-treated IPEC-J2 cells switch to a less aerobic metabolism, namely glycolysis *vs* OXPHOS, with respect to the control, but without affecting the mitochondrial respiration and the glycolysis parameters. The ATP production of VK2-treated IPEC-J2 cells is only increased at the lower VK2 concentration tested, namely 5 μM VK2. Indeed, the effect is corroborated by the Mito Stress Test which shows that the mitochondrial parameters are improved at 5 μM VK2 concentration tested. The VK2 structure resembles that of the CoQ_10_, a membrane-embedded electron carrier of mitochondrial electron transfer system (mETS) (Lenaz et al., 1999), even if it cannot substitute CoQ_10_ (Cerqua et al., 2019). VK2 can mimic the two structural components of CoQ_10_ which are crucial in OXPHOS, namely in the naphthoquinone core, which transfer electrons during the redox-cycle, and in the isoprenoid tail, which allows diffusion into the lipid bilayer. Recently both VK1 and VK2 were found to decrease the order and to increase fluidity in model membranes (Ausili et al., 2020). Therefore, these VKs may also act indirectly by facilitating the function of respiratory complexes through the inner mitochondrial membrane.

Short-chain quinones are known to be toxic, especially those containing 0–3 isoprene units, whereas CoQ_4_ displays only minimal toxicity (Takahashi et al., 2018). During mitochondrial respiration the VK2 might transfer electrons from complex I or complex II directly or indirectly to cytochrome *c*, according to the diffusion mode of shuttling electrons along the respiratory chain (Nesci and Lenaz, 2021). This VK2 role as electron transporter in the respiratory chain (Colpa-Boonstra and Slater, 1958) is still controversial. Accordingly, in spite of reports which rule out this possibility (Cerqua et al., 2019), in an *in vitro* model VK2 enhanced respiratory chain efficiency and contributed to building the electrochemical proton gradient (ΔμH^+^) by respiratory complexes that are exploited to generate ATP, similarly to CoQ_10_ (Vos et al., 2012). Our findings show that the effect on mitochondrial respiration could depend on VK2 concentration, namely the positive effect shown at 5 μM VK2 may become harmful at high concentrations. The VK2 structural properties are consistent with the stimulation of mitochondrial respiration without any effect on glycolysis. Consistently, low VK2 concentrations can favor an energy boost in IPEC-J2 cell activation, proliferation, and differentiation by increasing the OXPHOS capability to produce ATP.

Vitamer VK3 is the only one of the three forms of VK under study which exerts a negative effect on energy metabolism of IPEC-J2 cells. Accordingly, cell respiration is inhibited by both VK3 concentrations. The VK3-driven increase in H^+^ leakage through the inner mitochondrial membrane leads to the decrease in the ATP synthesis. However, uncoupling agents such as FCCP can stimulate the mitochondrial respiration of VK3-reated IPEC-J2 cells. The increase in non-phosphorylating substrate oxidation is typical of a decrease in ΔμH^+^, accompanied by production of reactive oxygen species and increase in oxygen consumption (Zorov et al., 2020). Conversely, we also detected in VK3-treated IPEC-J2 cells an inhibited mitochondrial respiration, as it happens in the presence of excess of uncouplers. Moreover, the negative effect on cell metabolism is underscored by VK3-driven enhancement of the ECAR-insensitive glycolytic activity in IPEC-J2 cells. However, VK3-treated IPEC-J2 cells can only rely on the glycolytic pathway to supply the energy required when their energy production mode shifts upon induction of signaling pathways of stress (Hung and Calkins, 2016).

The present study points out that the different VK vitamers tested promote different effects on IPEC-J2 cell bioenergetics. Accordingly, the chemical structure of the sidechain of VKs is confirmed to be crucial to produce the effects.

New roles for VKs in reducing risk of certain chronic diseases have been proposed in the last decades (Shearer et al., 2012). Consistently, the present work which shows for the first time how different VK forms and concentrations differently act on the bioenergetics of IPEC-J2 cell line contributes to deepening the knowledge on the varied roles of VKs in enterocytes, of which the IPEC-J2 cell line constitutes a fascinating model. The swine is increasingly used in translational research and drug development (Monticello and Haschek, 2016). Therefore, the benefits of a VK stimulation of ATP production in enterocytes underlines and confirms the relevance of gut microbiota, as VK producer, in maintaining a healthy gut and, as a cascade, in the prevention and/or treatment of diseases of the intestinal tract. Interestingly, since the last century (Krasinski et al., 1985) certain chronic gastrointestinal disorders were associated with VK deficiency. At present this connection is established with Crohn’s disease (Schoon et al., 2001), celiac disease (Djuric et al., 2007), and inflammatory bowel disease (Nowak et al., 2014). Pathological conditions are often associated with impaired mitochondrial bioenergetics (Nesci et al., 2021). Moreover, VK antagonists used in clinical therapy to prevent thromboembolism together with coadministration of other drugs can induce drug-drug interaction effects with VK malabsorption (Takada et al., 2015). Infants cannot obtain VK from the breast milk and have poor intestinal adsorption due to immature glut flora. Therefore, VK oral administration is the best way to prevent Vitamin K deficiency bleeding in infancy (Araki and Shirahata, 2020). It seems likely to postulate that an insufficient ATP production by enterocytes, related to a poor VK availability, also due to antibiotic treatments (Shirakawa et al., 1990), may represent, or contribute to, the biochemical bases of some gut pathologies.

## Data Availability Statement

The raw data supporting the conclusions of this article will be made available by the authors, without undue reservation.

## Author Contributions

CA and DLM performed the experiments. CA and CB analyzed the data. SN conceived the original idea. CB, MF, AP, and SN planned the experiments. SN supervised the project. AP and SN wrote the manuscript. CB, FT, and MF revised the text. MF acquired funding. All authors read and approved the manuscript.

## Conflict of Interest

The authors declare that the research was conducted in the absence of any commercial or financial relationships that could be construed as a potential conflict of interest.
